# Weight loss in conservative treatment of obesity in women is associated with physical activity and circadian phenotype: a longitudinal observational study

**DOI:** 10.1186/s13030-019-0163-2

**Published:** 2019-10-25

**Authors:** Eva Fárková, Jakub Schneider, Michal Šmotek, Eduard Bakštein, Jitka Herlesová, Jana Kopřivová, Petra Šrámková, Dita Pichlerová, Martin Fried

**Affiliations:** 1 National Institute of Mental Health, Sleep Medicine and Chronobiology, Topolová 748, 250 67 Klecany, Czech Republic; 2 Charles University – Third Faculty of Medicine, Prague, Czech Republic; 3 Czech Technical University in Prague, Faculty of Electrical Engineering, Prague, Czech Republic; 4 OB Clinic a.s., Praha, Prague, Czech Republic; 5 Pavel Kolář’s Centre of Physical Medicine, Prague, Czech Republic

**Keywords:** Obesity, Actigraphy, Circadian rhythm, Stability of the rhythm, Physical activity, Sleep

## Abstract

**Introduction:**

The study investigates the association between circadian phenotype (CP), its stability (interdaily stability - IS) and physical activity (PA) in a weight loss (WL) programme.

**Methods:**

Seventy-five women in WL conservative treatment (BMI ≥ 25 kg/m2) were measured (for about 3 months in between 2016 and 2018) by actigraphy.

**Results:**

We observed a difference in time of acrophase (*p* = 0.049), but no difference in IS (*p* = 0.533) between women who lost and did not lose weight. There was a difference in PA (mesor) between groups of women who lost weight compared to those who gained weight (*p* = 0.007). There was a relationship between IS and PA parametres mesor: p0.001; and the most active 10 h of a day (M10): *p* < 0.001 - the more stable were women in their rhythm, the more PA they have. Besides confirming a relationship between PA and WL, we also found a relation between WL and CP based on acrophase. Although no direct relationship was found for the indicators of rhythm stability (IS), they can be considered very important variables because of their close connection to PA – a main factor that contributes to the success of the WL programme.

**Discussion:**

According to the results of the study, screening of the CP and its stability may be beneficial in the creation of an individualized WL plan.

## Introduction

Epidemiological studies show that the prevalence of obesity is steadily rising. The Czech Republic is among the most obese nations worldwide (obesity prevalence greater than 25% in adult population). Intercountry comparable overweight and obesity (estimates from 2008) show that 66.1% of the adult population in the Czech Republic were overweight, 32.7% of whom were obese [1].

Overweight (=pre-obesity: BMI 25.0–29.9 kg/m2) and obesity (BMI ≥ 30.0 kg/m2) have been associated with elevated risk of many health complications [2, 3]. Obesity in women at the reproductive age carries high risk, not only for them but also for their offspring. These women are more often infertile and have increased maternal and pregnancy risks [4]. At birth, their children are more often obese and suffer from metabolic complications [5].

The literature suggests that circadian rhythms and sleep parameters are important in weight regulation and metabolism [2, 3, 6, 7]. It has been reported that people with different circadian preferences (i.e. morning vs. evening chronotypes) show distinct sleep/wake and eating habits and mental and physical performance. Specifically, morning/early chronotype (larks) spontaneously wake up at an early hour and find it difficult to stay up late in the evening; evening/late chronotype (owls) have the tendency to go to sleep late at night and have difficulty in getting up early. Most individuals occupy a scale position somewhere between these extreme types and can be described as neither, or intermediate (chrono)type [8]. The distribution of chronotypes in the general population approximates a Gaussian curve [9–12]. The most frequent differences are found in sleep regime, eating habits, psychological characteristics, the tendency to store fat, health-damaging behaviour, prevalence of lifestyle diseases (mainly metabolic, cardiovascular and immune diseases), and reactivity to seasonal changes [3, 7, 10, 13]. Evening chronotype women (late chronotype or “owls”) are more vulnerable to the pathogenesis of many types of health complications and often suffer from a higher risk of social jet-lag [14, 15]. Social jet-lag (SJL) quantifies the discrepancy that often arises between circadian and social clocks (i.e. stability of the circadian rhythm), which may result in chronic sleep loss [14]. SJL is associated with increased risk of developing sleep disorders, disruption of circadian rhythms and a higher prevalence of lifestyle and chronic diseases [14, 16, 17]. In most cases, this disruption is related to the sleep loss and, together, circadian and sleep disturbances are considered as risk factors for increased appetite, weight, blood pressure, and decreased glucose tolerance or immune response [3, 7].

## Materials and methods

### Aims

The aim of the project is to describe sleep patterns and circadian phenotype (CP) in a cohort of Czech women suffering from overweight or obesity, who participated in a conservative weight reduction programme at OB Clinic in Prague, Czech Republic. The main question was whether there was an association between the individual circadian phenotype, its stability including social jet-lag (i.e. mismatch between circadian and social time), and the outcome of the treatment. Furthermore, we were interested in whether and how sleep and activity parameters changed during the weight reduction programme (during the lifestyle change).

### Hypotheses

It was hypothesised that:

A, The earlier the circadian phenotype (lower acrophase) and the more stable the rhythm (higher interdaily stability (IS)), the more successful the treatment will be (larger BMI change (dBMI)).
B, Actigraphy parameters will change as a result of the weight change, resulting in better sleep (lower activity during the least active hours of a day (L5)) and more activity (larger activity during the most active hours of a day M10 and Mesor). We expect no changes in sleep duration.
C, The amount of physical activity will differ with regards to circadian phenotype and the stability of the rhythm. We expect more active participants (using M10 and mesor) to have a more stable rhythm (higher IS) and an earlier phenotype (lower acrophase).

### Study design

In this prospective, observational study, a group of obese and overweight women, who for the first time entered a conservative obesity treatment programme in this Clinic (participants chosen opportunistically), were monitored for up to 3 months between 2016 and 2018. The conservative weight reduction programme involved non-surgical interventions, including an individually tailored treatment plan, consisting of individual nutritional recommendations (a balanced diet plan with an emphasis on a low-calorie diet) and instructions for adherence to an adequate physical activity (depending on the severity of the health condition). At the same time, participants underwent regular individual sessions with a psychologist, with the aim of increasing their motivation and contributing to the success of the treatment. No pharmacological support or medication was offered to patients in the conservative treatment programme. Informed consent was obtained from all individual participants included in the study. The study protocol has been approved by the committees on human research of both institutes (ref. no. 112/15 and 22/2).

At the beginning of the treatment, the participants underwent anthropometrical measurements (height, weight, waist and hip circumference) and were screened for their chronotype and social jet-lag using subjective measures: Morningness-Eveningness Questionnaire (MEQ), which asks for individual preferred times for different activities [8], was used for chronotype estimation, and Munich Chronotype Questionnaire (MCTQ), which reports typical behaviour on work days and work-free days separately [18], was used for social jet-lag estimation. Upon entry, each participant was equipped with an actigraph to be worn on the wrist of the non-dominant hand continuously throughout the participation in the study. Long-term actigraphy was focused on the following parameters: acrophase and interdaily stability (IS), to determine the circadian phenotype and its stability, and further parameters to capture possible changes in physical activity (PA) and sleep habits during the treatment: total daily sleep duration, activity in the least active hours of a day (L5), activity in the most active hours of a day (M10) and overall activity (mesor). In further exploratory analyses, these parameters were tracked during the first and the last 14 days of the programme, depending on whether the participating women had lost weight or not. The average length of actigraphic measurement was 2.6 months (mean 79.6 days, range 32–101 days).

### Sample, inclusion and exclusion criteria

Out of the 120 addressed women who underwent the screening examination, a total of 92 subjects were actigraphicaly monitored. Inclusion criteria were: age 18+ (majority), BMI ≥ 25.0 kg/m2, not of post-menopausal age, no shift work, no pharmacologically treated psychiatric illness, provided complete data and written consent to participate in the study. From the monitored sample (*N* = 92) 17 women did not meet the inclusion criteria [19–21]: night shifts during the course of the study (*n* = 8), pharmacologically treated psychiatric illnesses (*n* = 5), post-menopausal age (*n* = 2). Two more participants were excluded due to an incomplete questionnaire or actigraphic data, resulting in 75 participants (mean age 36.5; SD 8.3, range 18–50) eligible for further analyses. The sample size was a result of power analysis (G*power), [22] conducted prior to the study (expected effect size 0.5, power 0.80, *p* = 0.05), which estimated a minimum of 64 women necessary.

To confirm that all participants were obese, rather than of robust constitution, the waist to height ratio (WHtR) was computed, and all women reached WHtR≥0.5. This value is considered to be the threshold for abdominal obesity in women, which represents a high risk for other health complications, especially cardiovascular and metabolic [23]. Based on weight loss (the value of BMI difference (BMI at the end minus BMI at the beginning of the study, dBMI), which we assumed to be a measure of success in the weight reduction programme, we divided our data set into three groups (weight loss, dBMI<− 0.7: weight gain, dBMI > 0.7: and no change). As weight may oscillate slightly during the menstrual cycle, weight changes of up-to + − 2 kg were considered as no change in status. A change of weight of up-to + − 2 kg at the average height (in our sample of 1.68 m) corresponds to change in BMI (dBMI) by + − 0.7 kg/m^2^.

### Actigraphy

Actigraphy is a widely used method for long-term monitoring of individual circadian rhythms and sleep timing in the individual’s private environment. In this study, the device (MindG actigraph by Mindpax.me) was worn on the wrist of the non-dominant arm and was set to collect activity counts in 30s epochs. Using base stations, placed at participants’ homes, the data were wirelessly transferred to a server, where they were stored for offline processing.

Both parametric and non-parametric features were used to extract features from the actigraphy signal. All feature extractions, apart from sleep detection, were performed on raw actigraphy data, using a custom toolbox in the Matlab environment (MATLAB 2015b, The MathWorks, Inc., Natick, Massachusetts, United States).

#### Parametric features

The parametric approach is represented by the cosinor analysis. Using this method, the cosine function with a fixed period (24 h) was fitted to the raw actigraphy data and the following parameters were evaluated: mesor (midline-estimating statistics of the rhythm - offset), and acrophase (phase shift). The mesor is proportional to the overall mean activity during the 24 h and is therefore affected by the level of exercise. The acrophase can be regarded as an objective measure of chronotype and can be used to evaluate its possible change [24–26]. The acrophase was also used to exclude data where travel between time zones occurred.

This study aimed to follow changes of these parameters and therefore the cosine functions were fitted on a two-week (14-day) window, which always overlapped by 13 days (83%) - one value per day.

#### Nonparametric features

The mean activity during the least active five-hour period (L5) and the most active ten-hour period (M10), [27] were computed. In addition, we computed the interdaily stability (IS), based on chi-square periodogram [28], to depict synchronisation with the light-dark cycle. The IS was computed from a 14-day window, based on 30-min resampled data [29]. The L5 parameter, calculated on a daily basis, was used to represent quality of sleep (independent of external sleep detection). The M10 parameter was used to represent the daytime daily activity.

In addition, total daily sleep duration, computed by the Mindpax system, was included. Daily sleep was defined as all detected sleep periods that occurred during 1 day (24 h), including night sleep as well as all naps. Detected non-wear time periods (when the device was removed) were excluded from the analyses.

### Questionnaires

For internal control and comparison with actigraphic parameters, women were screened for chronotype and rate of social jet-lag using commonly used questionnaires.

Participants were screened for their chronotype using the Morningness-Eveningness Questionnaire (MEQ) developed by Horne and Östberg (1976). The MEQ contains 19 multiple-choice questions, enquiring about individually preferred times for different activities. MEQ score ranges from 16 to 86, with lower values indicating more an evening chronotype. The MEQ score was used to control the acrophase actigraphic parameter (more in chapter 2.7).

The Munich Chronotype Questionnaire (MCTQ) designed by Roenneberg and his team (2003) was used because of its ability to measure the rate of social jet-lag (SJL). The rate of social jet-lag quantifies (in hours and minutes) the discrepancy between circadian and social clocks (the difference between hours of sleep on free days and working days), which can lead to chronic sleep loss [14]. The SJL score was used to control the actigraphic parameter interdaily stability (more in chapter 2.7).

Czech versions of all questionnaires, which were validated by a double-reverse translation from the originals, were used. Permission from the authors was obtained for their translation and use. Reliability and validity of translated versions were not re-evaluated, but the acceptability of MEQ translation (Cronbach’s alpha = 0.87) has already been demonstrated in a previous study [30]. Similar data for the translation of MCTQ are unavailable.

### Determining the circadian phenotype

Both objective (acrophase for the circadian phenotype and IS for its stability during work- and free-days) and subjective circadian parameters were used in this study. Based on previous studies [24–26, 31], objective actigraphy data were selected for further analyses.

A newly created scale, based on actigraphy-measured acrophase was used to determine individual’s circadian phenotype. The acrophase corresponds to the time of day when physical activity culminates in an individual. The lower the value of the acrophase, the earlier the peak activity. This indicator is well related to the individual’s circadian rhythm or phenotype. Acrophase values in our data set correlated with the values obtained from MEQ (*r* = − 0.612; *p* < 0.001), which is similar to values described in a Korean validation study, where a significant negative association between Korean MEQ score and the timing of activity acrophase was found [25]. Also comparison of acrophase locations between morning active and evening active individuals (based on shortened MEQ) revealed that the peaks in the oral temperature and heart rate and random number speed occurs earlier in morning active individuals [24]. Furthermore, Lee and his team (2014) found the mean activity acrophase of the evening type group was nearly 2 h later than that of the morning type group. This difference was even greater on free days than on work days [25].

MEQ questionnaire, on the other hand, reflects the subjective preference of a certain time of day for different types of activities, including sleep, sporting activities, or mentally challenging tasks, but this preference may not be entirely consistent with the reality being experienced. Moreover, MEQ is a subjective tool, more susceptible to making an error in defining the chronotype. There is also a high risk of being affected by current mood and fatigue when completing the questionnaire.

Therefore, the set was divided into 3 categories according to the participants’ acrophase – early phenotype (EP = 17), middle phenotype (MP = 48) and late phenotype (LP = 10): see Table 1. The cut-off points for these 3 groups were: early phenotype, acrophase < 13.9: middle phenotype, acrophase 13.9–16.0: and late phenotype, acrophase > 16.0 (as middle, we have noted 50% of individuals around the median).

**Table 1:** Objectively defined circadian phenotype and interdaily stability distribution

	n	Acrophase	BMI at begining (kg/m^2^)	dBMI (kg/m^2^)	Age	Mesor (diff. the first and last 14 days)	IS	Social jet-lag	Chronotype
	10	Late phenotype	34.4 ± 10.4	0.2 ± 0.8	27.6 ± 6.0	−42.2 ± 144.2	0.5 ± 0.1	1.5 ± 1.1	44.9 ± 10.1
	48	Middle phenotype	34.9 ± 6.1	−1.1 ± 1.4	36.9 ± 7.9	− 15.4 ± 77.0	0.6 ± 0.1	1.3 ± 0.8	52.8 ± 7.2
	17	Early phenotype	32.8 ± 6.5	− 0.7 ± 1.8	40.7 ± 7.0	2.3 ± 72.0	0.6 ± 0.1	1.3 ± 1.0	59.8 ± 6.7
Total	75		34.3 ± 6.8	− 0.8 ± 1.5	36.5 ± 8.3	− 14.9 ± 87.1	0.6 ± 0.1	1.3 ± 0.9	53.4 ± 8.6
		sig. & type of test	*p* = 0.560 (*F* = 0.585)	***p*** **= 0.049** (*F* = 3.146)	***p*** **< 0.001** (*F* = 9.629)	*p* = 0.749 (χ2 = 0.578)	*p* = 0.624 *F* = 0.475)	*p* = 0.657 (*F* = 0.422)	***p*** **< 0.001** (*F* = 10.747)
	n	IS	BMI at begining (kg/m^2^)	dBMI (kg/m^2^)	Age	Mesor (diff. The first and last 14 days)	Acrophase	Social jet-lag	Chronotype
	33	Stable	33.9 ± 5.5	−0.6 ± 1.3	37.7 ± 7.6	9.9 ± 69.6	14.6 ± 0.9	1.0 ± 0.9	53.9 ± 8.1
	34	Slighty unstable	34.5 ± 6.9	−1.1 ± 1.7	36.1 ± 9.3	−27.5 ± 94.6	14.6 ± 1.0	1.6 ± 0.8	53.8 ± 9.0
	8	Unstable	35.6 ± 11.3	− 0.5 ± 1.3	33.0 ± 6.0	−64.4 ± 96.9	15.3 ± 1.1	1.7 ± 0.9	49.0 ± 9.0
Total	75		34.3 ± 6.8	− 0.8 ± 1.5	36.5 ± 8.3	− 14.9 ± 87.1	14.7 ± 1.0	1.3 ± 0.9	53.4 ± 8.6
		sig. & type of test	*p* = 0.821 (*F* = 0.198)	*p* = 0.533 (χ2 = 1.260)	*p* = 0.348 (*F* = 1.072)	*p* = 0.106 (*F* = 2.321)	*p* = 0.382 (*F* = 0.977)	*p* = 0.066 (*F* = 2.828)	*p* = 0.405 (*F* = 0.916)

Circadian phenotype (CP) distribution (based on the acrophase) and mean BMI at the beginning of the treatment, the mean dBMI (change of BMI during actigraphy measurement), mean age, chronotype and social jet-lag, mean mesor difference between the first and last 14 days of measurement, means intradaily stability (IS) from the whole measurement period. The late phenotype is the most likely to gain weight (mean 0.2 kg/m^2^), while the middle phenotype lost weight of 1.1 kg/m^2^ on average and the early phenotype lost weight of 0.7 kg/m^2^ on average. A significant relationship was found between acrophase and change in BMI (dBMI) (*F* = 3.146, *p* = 0.049) and between acrophase and age (*F* = 9.629, *p* < 0.001). Participants were divided according to the stability of their circadian rhythm (IS) and the respective means of the same variables. Statistical analyses showed that there was no significant relationship between dBMI and IS (χ2 = 1.260, *p* = 0.533). At the same time, the link between acrophase and chronotype was confirmed

Similarly to MEQ and acrophase, we chose IS as an objective equivalent of SJL to use in further analyses. Despite the fact that IS is not a perfect equivalent of SJL, it is the only actigraphy-based measurement related to it referring to stability of circadian phenotype. Furthermore, there is no standardized form for categorization of social jet-lag and IS accepted in the literature. Studies have shown that IS is directly related to rhythm amplitude and light exposure [26, 31]. IS can quantify a mismatch between circadian and social time and thus it describes the degree of resemblance between the activity patterns on individual days; it ranges from 0 to 1 and may typically be about 0.6 [27, 28, 31]. The closer to 1, the more stable is the rhythm over the evaluated period. The division (corresponding to SJLrel values, *r* = − 0.29;, *p* = 0.011), was based on mean and median values (0.56, a value similar to other studies [27, 28, 31]), which corresponded to a SJL of about 1h20min. Values of SJL were paired with the values for IS, and based on the work of Islam et al., a cut-off for IS was created based on SJL values (< 1 h, 1 to < 2 h or ≥ 2 h) [32], resulting in 3 categories: stable (S = 33), slightly unstable (SU = 34), unstable (U = 8), was used for analysis (Table 1). The cut-off points for these 3 groups were: stable, IS 0.58–1: slightly unstable, IS 0.44–0.57: unstable, IS 0–0.43.

### Statistical analysis

Data were evaluated using IBM SPSS Statistics 23: Pearson correlations were used to determine the relationship between selected variables. ANOVA, independent-samples t-test and its non-parametric alternative (Kruskal-Wallis) were used where the criterion of normality was not met to calculate the differences between the groups of participants. Generalized linear model was used to control for baseline BMI and age as confounding variables. The LSD post-hoc test was used for multiple comparisons to determine specific group differences. Actigraphic measures were processed using Matlab 2015b.

## Results

### Collected data

**Fig. 1:**
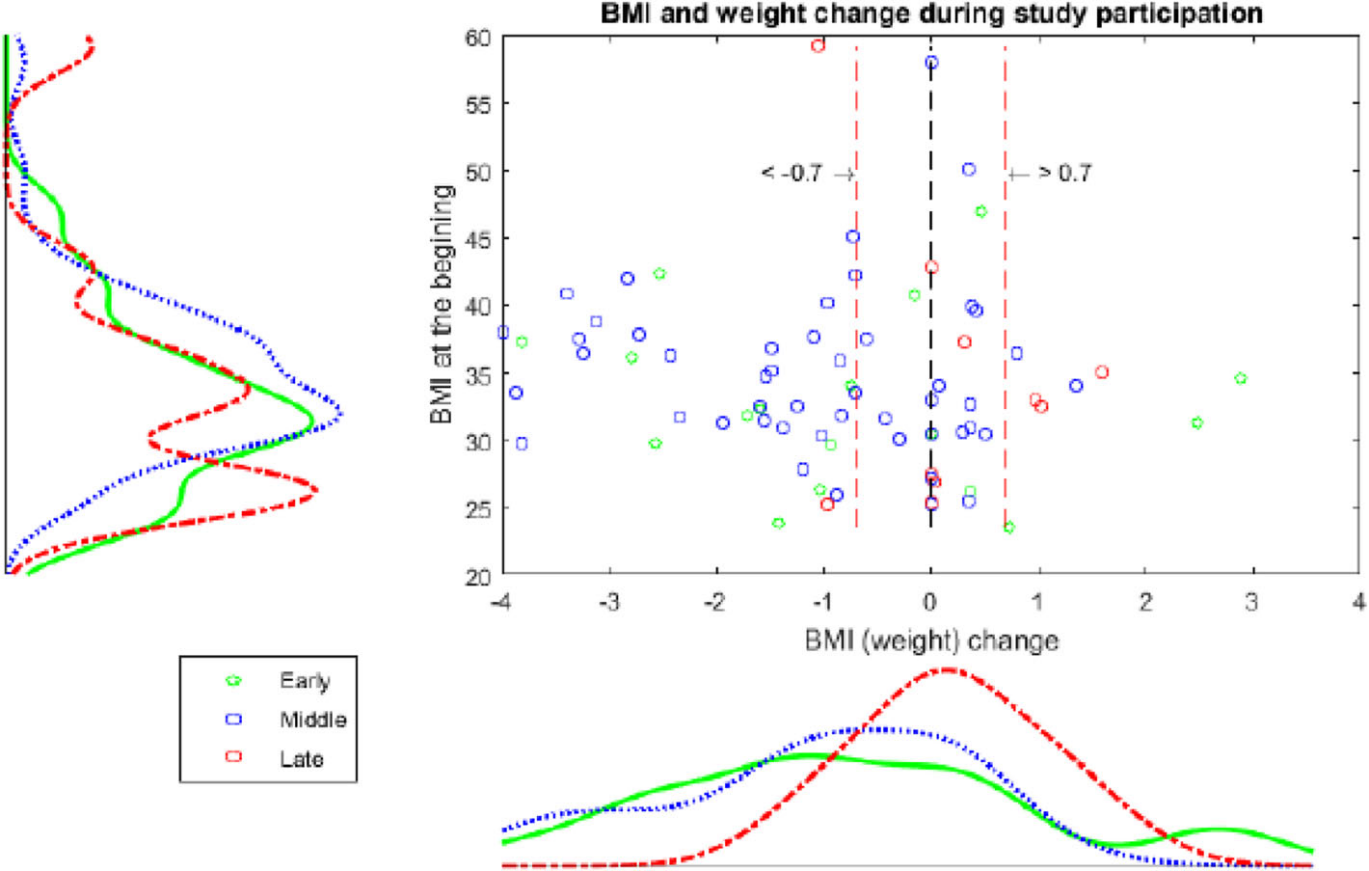
Sample proportions by BMI and weight change during study participation. The red lines indicate the area including subjects whose BMI changed by less than 0.7 kg/m2 above or below the original BMI

Based on the initial BMI at study entry (see Fig. 1), nearly 21% of the women were overweight (BMI ≥ 25 kg/m^2^), and 79% were obese (BMI ≥ 30 kg/m^2^). After completing the conservative weight loss programme, 41 participants lost weight, 8 participants gained weight, and, in 26 participants, the BMI values remained unchanged (i.e. the change was lower than 0.7 kg/m^2^, Fig. 1).

### Results of data analysis

No relationship was observed between age and dBMI (*r* = − 0.063; *p* = 0.591), nor between age and BMI (*r* = − 0.021; *p* = 0.859). In addition, no age difference was found between groups by dBMI (*F* = 0.977; *p* = 0.381) using general lineal model. However an association between age and acrophase (categorical) was tested and found to be significant (*F* = 9.629, *p* < 0.001), which is why age was used as a confounding variable, along with baseline BMI, in further analyses.

With regards to our research hypotheses, significant differences in dBMI were found between groups of participants divided by their acrophase (*F* = 3.146; *p* = 0.049). Afterwards, a group post-hoc test (LSD test) was performed for differences in dBMI between the acrophase categories. There was a significant difference between the groups of women with late phenotype, which is less easy to lose weight, compared to those with middle phenotype (*p* = 0.012), see Table 1. However, there were no differences in dBMI between IS categories (χ2 = 1.260; *p* = 0.533), meaning that possible misalignment between social and biological clocks had no effect on BMI change.

**Fig. 2:**
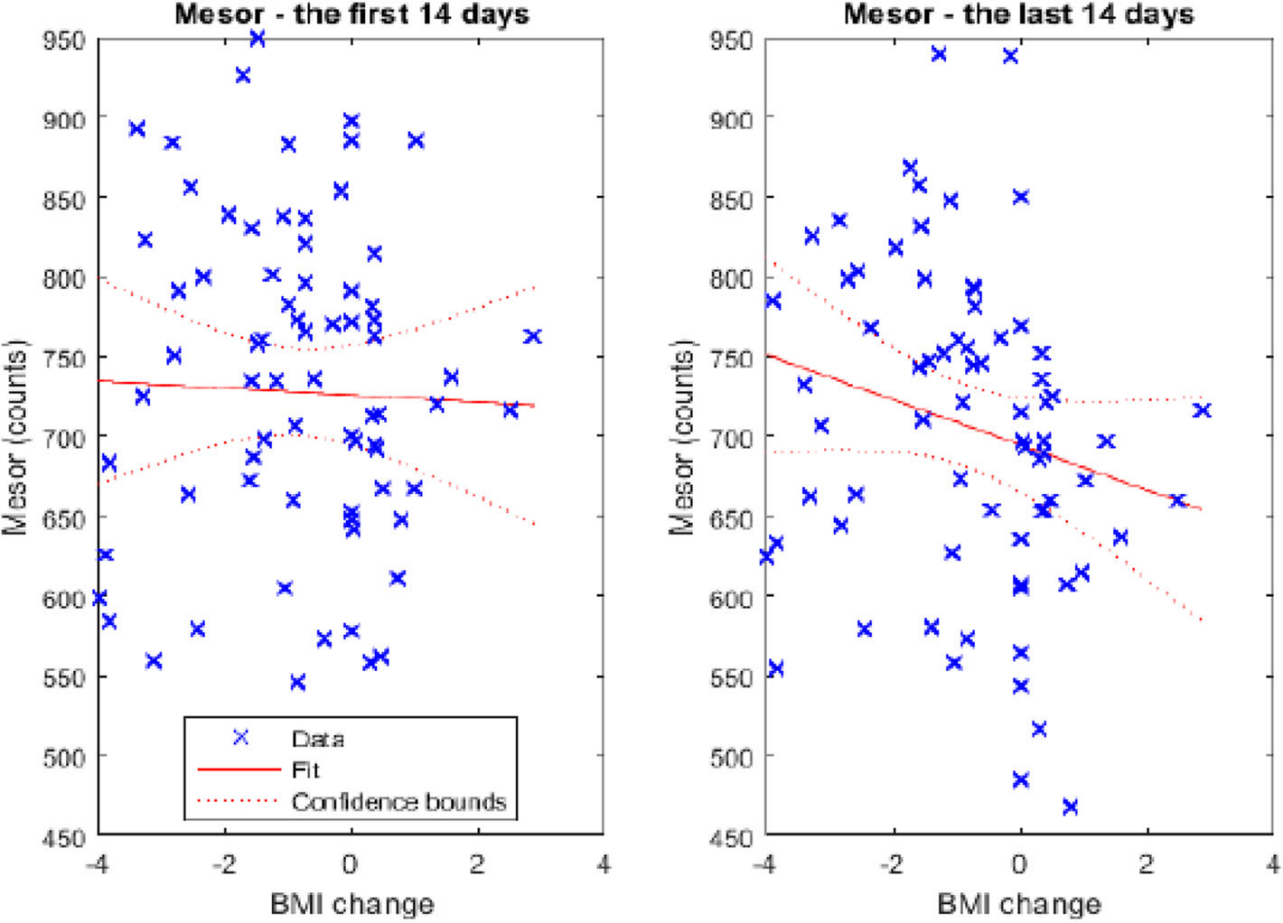
Level of total average activity (mesor) at the beginning and at the end of the programme (the first and last 14 days of measurement); all women had approximately the same level of total average activity (mesor) at the beginning, but at the end of participation in the programme, significant changes were observed

In terms of weight change in the programme, no significant differences were found among the 3 groups by dBMI in the maximum daily activity M10 (*F* = 1.850; *p* = 0.165), in sleep activity L5 (*F* = 0.545; *p* = 0.582) or in total sleep duration (*F* = 0.428; *p* = 0.653). However, significant differences were found in terms of the daily physical activity (mesor). While, at the beginning of the programme (the first 14 days of measurement), all women had approximately the same level of overall activity (mesor), at the end of participation in the programme, significant changes were observed (*F* = 3.821; *p* = 0.027). After running post-hoc tests (LSD test) for differences in mesor between the first and the last 14 days in the study, a significant difference between the groups of women who lost weight compared to those who gained weight (*p* = 0.007) was found. Women who gained weight experienced a decline in their level of overall activity during the course of the treatment, while those that lost weight kept their level of overall activity unchanged (see Fig. 2).

Finally, it has been shown that the parameters referring to the activity (mesor and M10) in the set are not related to the acrophase (*F* = 0.045, *p* = 0.956 for mesor and *F* = 0.337, *p* = 0.715 for M10). Conversely, differences in mesor (*F* = 8.461; p0.001) and M10 (*F* = 11.035; *p* < 0.001) between groups of participants divided by their IS were found. Post-hoc tests revealed a significant difference between the groups of women with stable phenotype, compared to those with slightly unstable (*p* = 0.001 for mesor and *p* < 0.001 for M10) and unstable phenotype (*p* = 0.003 for mesor and *p* < 0.001 for M10). These findings indicate that, regardless of acrophase, women with a stable circadian rhythm are significantly more active than women who do not have a stable phenotype (Table 1).

## Discussion

The aim of the current study was to investigate the relationship between circadian phenotype (acrophase) and stability of circadian rhythms (IS) in relation to BMI change (dBMI) during conservative non-surgical treatment of obesity [33] in Czech women.

According to our first hypothesis, women with early phenotype (early acrophase) with more stable rhythm (i.e. high IS), were expected to be more successful in losing weight. The hypothesis was partially confirmed, as we found that women with middle phenotype were more successful in losing weight than were those with late phenotype. This is in line with a study by Raynor et al. (2018), where slower weight loss during a diet intervention was observed in late types, defined by a later food intake [34]. No differences in dBMI between IS categories were found, so it can be assumed that rhythm stability does not affect weight loss. In contrast to our results, some existing studies suggest a positive association between subjective social jet-lag (i.e. mismatch between circadian and social time) and BMI [16] On the contrary, Malone et al. reported no relationship between chronotype and BMI [35], which corresponds to other findings [36, 37] and is in line with our results. Instead, thanks to having an objective measure of participants’ phenotype, this study demonstrated that there was a significant association between changes in BMI during the treatment of obesity and the circadian phenotype (CP), where early CPs suggest a higher chance of success in a weight-loss programme. It is necessary to take into account the fact that acrophase and IS can vary with the age [10]. However, our dataset contained only females in productive age (no longer students and not yet retired), assuming that their circadian rhythm had already been stabilized [14]. For this reason, acrophase was counted as confounding variable for age and dBMI analysis.

Compared to other studies, this study benefits from the strengths associated with long-term actigraphy monitoring. Several previous studies, which evaluated diet type and amount of exercise with regard to BMI change confirmed the obvious influence of diet and physical exercise on weight loss [36, 38, 39]. The vast majority of studies with a similar theme are concerned with a specific type and amount of exercise with regard to BMI change. During the conservative treatment in this study, the participants were educated and instructed to change their diet plans and were individually recommended to be physically more active, depending on the severity of their obesity [33, 40]. Despite the fact that the PA regime was not standardized or controlled during, or before, the course of the treatment, we had an objective, actigraphic measure that reflects the total motor activity of the participants (mesor). At the beginning of the weight-loss programme, all participants had approximately the same level of activity (mesor), which corresponds to the results of the study by Vitale et al. where the authors did not find any difference in mesor values between CP [41]. However, only those who lost weight (or did not change their weight) had the same level of activity at the end of their participation in the conservative treatment of obesity. In women who gained weight a decrease in activity was observed.

Together, these results suggest that weight loss does not depend on the sleep duration or on IS, but rather on the CP and maybe mostly on the ability to strictly follow a PA regime (regardless of type) and recommended lifestyle changes. Significant difference was found between IS (but not for acrophase) and activity parameters. Therefore, in terms of losing weight, it also seems important to live in accordance with one’s self-circadian phenotype, having a stable rhythm, rather than being a morning or evening chronotype person. It has to be noted, however, that the effect size of the observed association between weight loss and CP was small and we therefore conclude, that CP is rather a partial contributor to weight loss success among other apparent and stronger factors discussed above.

It is also important to acknowledge several limitations in the present study. Firstly, the sample was drawn from a specific population of women attending the WL programme, thus the authors are aware that these conclusions cannot be generalized for the whole population of obese or overweight people. Also, the dataset did not contain healthy controls. Results that would include healthy controls, participants working different work-shift regimes, participants with psychiatric comorbidities, or women with post-menopausal status may yield different results. However, the authors believe that thanks to this kind of sampling they were able to rule out possible interactions of disturbed sleep-wake cycle due to irregular regime, medication’s effect, and the effect of potential age-associated hormonal changes.

Secondly, the distribution of individuals in the age categories was not normal. However, age-related statistical analyses did not confirm a significant link between age and weight loss. The only significant relationship was found between age and acrophase, which was previously confirmed in a study by Tankova et al., suggesting that acrophase reflects one’s circadian phenotype, moving towards the morning type with increasing age [42], similarly to MEQ scores.

Thirdly, this was not a gender-balanced study. A set of only women was deliberately chosen because of the hormone-controlled differences in circadian rhythms that may be sex-dependent. According to research on objective sleep parameters, only post-menopausal women show statistically significant differences in the setting of sex hormones that can interfere with the control of circadian rhythms and can also change sleep parameters [19]. Although menopausal or post-menopausal women were excluded, it should be noted that hormonal changes might have already started in some study participants.

Our results suggest that the success in weight loss may be influenced by the circadian phenotype (acrophase), but not by IS. One of the benefits of the study is that objective and subjective (for comparative contrast) circadian phenotype measurements were used. In addition, it has been shown that physical activity plays a major role in weight loss alongside acrophase, but stability of the CP may also play a role. Motivation and the ability to follow lifestyle changes strictly on a long-term basis are important prerequisites for weight loss. In comparison to some other studies [36, 37], this study does confirm the relationship between weight loss and circadian phenotype, using the objective method of identifying the circadian phenotype.

## Conclusion

Understanding the context of chronotypes and how exactly circadian rhythms function in combination with individual habits in relation to health issues may assist in developing new treatment strategies adjusted to individual biological clocks to create targeted preventive and therapeutic methods for acute and chronic diseases, as the vast majority of them are related to our circadian rhythms. The topic is now coming to the forefront of scientific as well as clinical interest, as it has been found that it is very important to consider individual settings of circadian rhythms in the treatment of many diseases [43]. The present study extends the knowledge relevant especially to clinical practice. Physical activity has been shown to have a major impact on whether the weight loss program is successful, and that the rate of physical activity may be dependent on the stability of the circadian rhythm. In addition, it has been shown that the time of acrophase, i.e. the individual circadian phenotype, can affect the success of weight loss therapy. It follows that the screening of circadian phenotype and its stability may be beneficial in the creation of an individualized weight loss plan.

## Data Availability

Study data are available from the corresponding author on reasonable request.
